# Supplementary material for: Abnormal Social Interactions in a Drosophila Mutant of an Autism Candidate Gene: Neuroligin 3
Source: Int J Mol Sci. 2020 Jun 29;21(13):4601. doi: 10.3390/ijms21134601 (PMC7370170; doi:10.3390/ijms21134601)
Supplement: Supplementary file 1 [file ijms-21-04601-s001.pdf]

Supplemental Data - Abnormal social interactions in a *Drosophila* mutant of an autism candidate gene: *neuroligin 3*

Ryley T. Yost[1#], J. Wesley Robinson[1#], Carling M. Baxter[2], Andrew M. Scott[2], Liam P. Brown[1], M. Sol Aletta[1], Ramtin Hakimjavadi[1], Asad Lone[1], Reuven Dukas[2], Robert C. Cumming[1], Brian Mozer[3] and Anne F. Simon[1,*]

[1] Western University; [2]McMaster University; [3] Office of Research Integrity, OASH, Rockville, MD
* Correspondence: asimon28@uwo.ca; Tel: +1-519-661-2111X80084 (ON, Canada)
[#] equal contribution

Results

Low nlg3 transcript level is confirmed by ddPCR

For absolute quantification of *nlg3* transcript level, ddPCR was performed on cDNA generated from head tissue. The *nlg3* transcript was expressed at an extremely low level in comparison to the stably transcribed reference gene, *ribosomal protein L32* (*rpl32*). The mean total number of droplets generated for the ddPCR run was 10,548. The mean concentration of input cDNA present was 21 copies/µL for *nlg3*, and 2,520 copies/µL for *rpL32* (Supplemental Figure S1). To account for the dilution factor of input cDNA, the copy number per 20 µL reaction was multiplied by 10 for *nlg3* and by 100 for *rpL32* to estimate the initial copy number of the gene target in undiluted cDNA. The transcript abundance was approximately 1000-fold lower than that of rpL32, thereby confirming low copy number of the *nlg3*.

Material and Methods

Western Blot

Refer to materials and methods in the main text for Western blots methods.

Droplet digital PCR

To quantify *nlg3* in *D. melanogaster*, a droplet digital PCR (ddPCR) assay was developed using novel primers targeting exons 11–13 (F: 5'-ACTGGTCCAACTTTGTGCGA-3', R: 5'-GCTTCGGCTTGGTGTCAAAA-3'). Quantification of the housekeeping gene *rpl32* was performed using primers reported previously [1]. Total RNA was isolated from the heads of 50 four-day old mixed-sex *D. melanogaster* by TRIzol™ Reagent (Invitrogen, Waltham, Massachusetts, USA) isolation. RNA was quantified by spectrophotometry using a NanoPhotometer P300 (Implen Inc., Westlake Village, CA, USA), genomic DNA was removed using the TURBO DNA-free™ Kit (Invitrogen, Waltham, Massachusetts, USA), and cDNA was synthesized using the iScript™ cDNA Synthesis Kit (BioRad, Mississauga, ON, Canada). PCR amplification of *nlg3* and *rpl32* [1] was performed in triplicate 20 µL reactions, using QX200™ ddPCR™ EvaGreen® Supermix (BioRad, Mississauga, ON, Canada), 100 nM each of forward and reverse primers, and 1 µL diluted input cDNA (1:10 for *nlg3*, 1:100 for *RpL32*). PCR was performed under the following thermocycling conditions, with a ramp rate of 2°C / s for each step: Initial denaturation for 5 min at 95°C; 45 cycles of denaturation (30s, 95°C), annealing and extension (30 s, 58°C); signal stabilization for 5 min at 4°C then 5 min at 90°C. Droplets were analyzed using the QX200™ Droplet Reader with QuantaSoft software (v1.7.4.0917, BioRad, Mississauga, ON, Canada). The initial copy number of the gene target per 20 µL reaction (initial copy number per 1 µL diluted input cDNA) was calculated by the software.

References

1. Ling D.; Salvaterra P. M., Robust RT-qPCR data normalization: Validation and selection of internal reference genes during post-experimental data analysis. *PLoS ONE* **2011,** 6, e17762.

Supplemental Figures

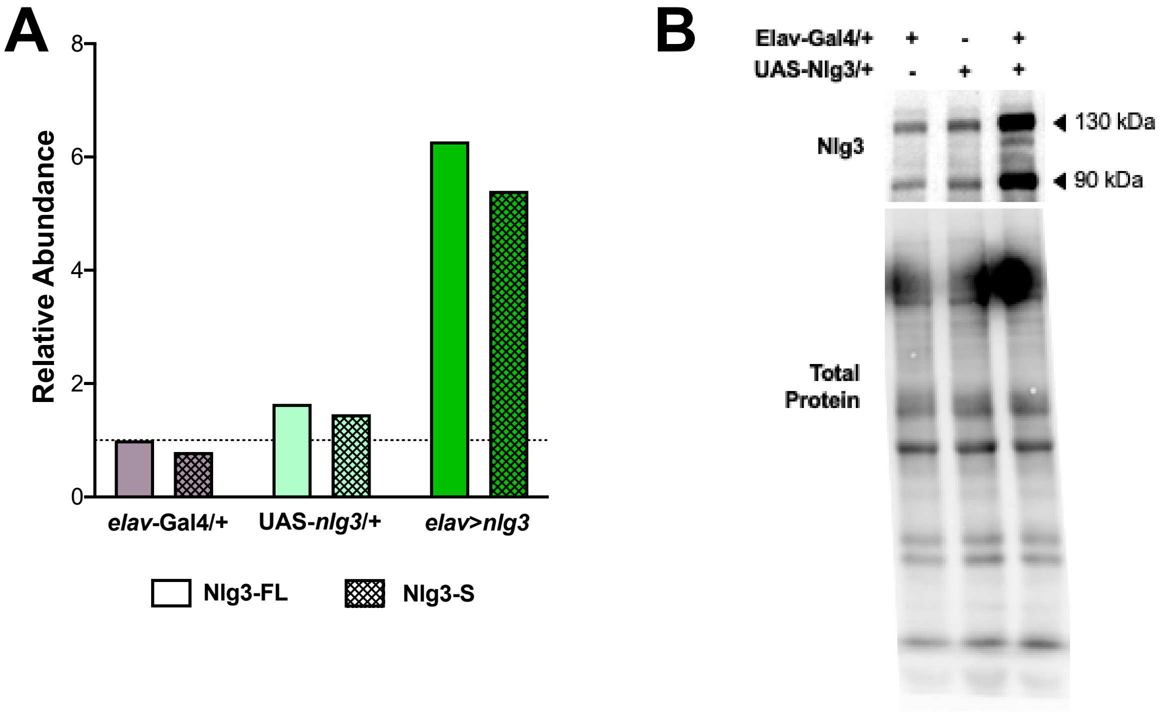

Supplementary Figure S1. Western blot confirmation of Nlg3 overexpression. (A) Nlg3 protein abundance in *elav>nlg3*. All treatments are displayed as relative abundance and normalized to Nlg3-FL in the *elav*-Gal4/+ control. n=1 for all treatments. **(B)** Representative Western blot of anti-Nlg3 immunoreactivity is displayed for each driver and the overexpression for Nlg3 for both protein isoforms. All treatments are normalized to total protein observed using BioRad stain-free technology. Each treatment is mixed sex with 10 males and 10 females. Of note: Nlg3 levels were not different in *elav*-Gal4/+ compared to Canton-S (data not shown).

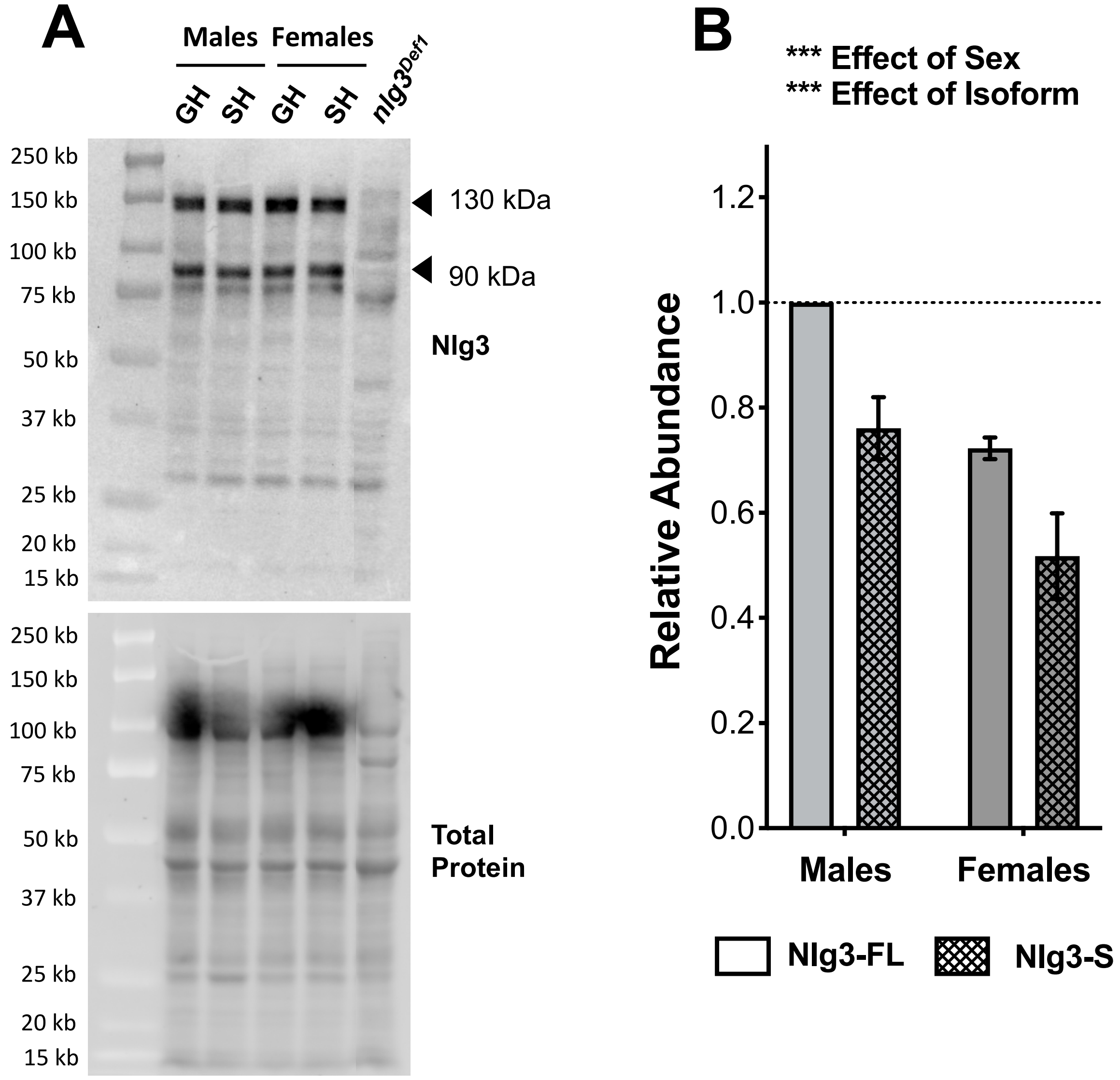

Supplementary Figure S2. Representative whole blot images with molecular weight markers and total protein (A) for group and single housed Cs (Figure 5C, D) including a male to female comparison in protein abundance (B). (A) Western blot of anti-Nlg3 is displayed (top image), along with total protein (bottom image). Lane 1 in each image is the molecular weight marker. Each treatment is mixed sex with 10 males and 10 females. **(B)** Mean protein abundance ± s.e.m. in male and female Cs. Group housed female Cs had lower protein levels than group housed males (two-way ANOVA: $F_{1,12}=18.81, P=0.001$) and both sexes had lower Nlg3-S than Nlg3-FL (two-way ANOVA: $F_{1,12}=25.90, P=0.0003$). All treatments are displayed as relative abundance to male Nlg3-FL. n=4 for all treatments.

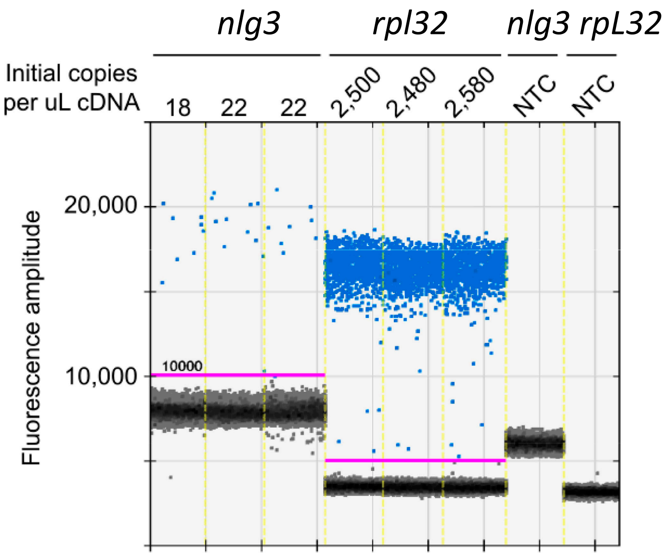

Supplementary Figure S3. Scatter plot of a ddPCR assay displaying fluorescence amplitude and original cDNA copy number for *nlg3* and *rpl32* in Cs. Treatments are separated by a yellow line. Purple line represents the fluorescence threshold. Above the threshold is a positive droplet for the cDNA of interest. Three technical replicates for *nlg3 and rpl32* and one replicate for the no template controls were conducted. NTC: No template control.

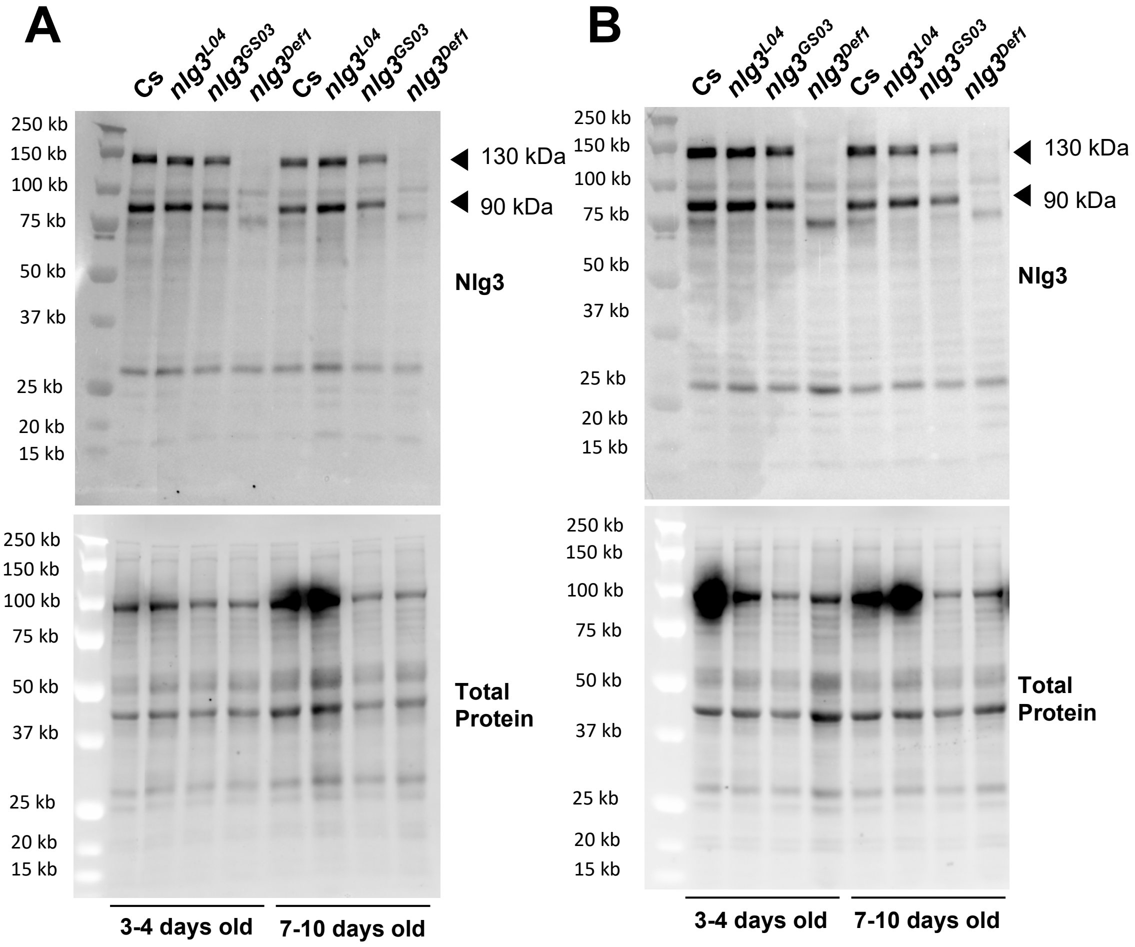

Supplementary Figure S4. Representative whole blot images with molecular weight markers and total protein for *nlg3* mutants (Figure 1B-E) for males (A) and females (B). Western blot of anti-Nlg3 is displayed for Cs and *nlg3* mutants (top image) and total protein (bottom image). Lane 1 in each image is the molecular weight marker. Each treatment is mixed sex with 10 males and 10 females.
